# Do genetic risk scores for childhood adiposity operate independent of BMI of their mothers?

**DOI:** 10.1038/s41366-021-00869-4

**Published:** 2021-05-28

**Authors:** Lam O. Huang, Camilla S. Morgen, Lars Ängquist, Ellen A. Nohr, Tuomas O. Kilpeläinen, Torben Hansen, Thorkild I. A. Sørensen, Theresia M. Schnurr

**Affiliations:** 1 Novo Nordisk Foundation Center for Basic Metabolic Research, Faculty of Health and Medical Sciences, University of Copenhagen, Copenhagen, Denmark; 2 Clinical Epidemiology Group, Steno Diabetes Center Copenhagen, Copenhagen, Denmark; 3 National Institute of Public Health, University of Southern Denmark, Copenhagen, Denmark; 4 Research Unit for Gynaecology and Obstetrics, Department of Clinical Research, University of Southern Denmark, Odense, Denmark; 5 MRC Integrative Epidemiology Unit at the University of Bristol, Bristol, UK; 6 Section of Epidemiology, Department of Public Health, Faculty of Health and Medical Sciences, University of Copenhagen, Copenhagen, Denmark

**Keywords:** Risk factors, Epidemiology, Genetics

## Abstract

**Objectives:**

Genetic predisposition and maternal body mass index (BMI) are risk factors for childhood adiposity, defined by either BMI or overweight. We aimed to investigate whether childhood-specific genetic risk scores (GRSs) for adiposity-related traits are associated with childhood adiposity independent of maternal BMI, or whether the associations are modified by maternal BMI.

**Methods:**

We constructed a weighted 26-SNP child BMI-GRS and a weighted 17-SNP child obesity-GRS in overall 1674 genotyped children within the Danish National Birth Cohort. We applied a case-cohort (*N* = 1261) and exposure-based cohort (*N* = 912) sampling design. Using logistic regression models we estimated associations of the GRSs and child overweight at age 7 years and examined if the GRSs influence child adiposity independent of maternal BMI (per standard deviation units).

**Results:**

In the case-cohort design analysis, maternal BMI and the child GRSs were associated with increased odds for childhood overweight [OR for maternal BMI: 2.01 (95% CI: 1.86; 2.17), OR for child BMI-GRS: 1.56 (95% CI: 1.47; 1.66), and OR for child obesity-GRS 1.46 (95% CI: 1.37; 1.54)]. Adjustment for maternal BMI did not change the results, and there were no significant interactions between the GRSs and maternal BMI. However, in the exposure-based cohort design analysis, significant interactions between the child GRSs and maternal BMI on child overweight were observed, suggesting 0.85–0.87-fold attenuation on ORs of child overweight at higher values of maternal BMI and child GRS.

**Conclusion:**

GRSs for childhood adiposity are strongly associated with childhood adiposity even when adjusted for maternal BMI, suggesting that the child-specific GRSs and maternal BMI contribute to childhood overweight independent of each other. However, high maternal BMI may attenuate the effects of child GRSs in children.

## Introduction

Genetic determinants are known to affect childhood adiposity, measured by body mass index (BMI) as a quantitative trait or its upper extreme, overweight or obesity [1–4]. Numerous genes associated with monogenic (syndromic and nonsyndromic) and polygenic childhood obesity have been identified [5]. The identification of polygenic determinants of BMI variation and obesity risk in children started with individual genome-wide association studies (GWAS) [6], followed by meta-analyses of GWAS studies with ever-growing sample size [7–11]. These meta-analyses efforts have identified overall 27 common genetic variants for childhood BMI [9, 11], explaining around 3.6% of BMI variance in children [11], as well as 20 genetic variants associated with childhood obesity as a binary outcome [8, 10].

There is a large overlap between adiposity-associated genetic variants identified in adults and children. Among the 27 child BMI SNP, there are 22 SNPs that are either the same as or in high LD with SNPs identified for adult BMI [12]. Similar, among the 20 childhood obesity SNPs, there are 17 SNPs, that are either the same as or in high LD with SNPs identified for adult BMI [12]. This indicates that child adiposity and adult adiposity have shared genetic etiology. On the other hand, a few of the variants (five childhood BMI SNPs and three childhood obesity SNPs) that were identified in GWAS in children were not identified in GWAS of adult BMI [12], highlighting that there are adiposity-associated genetic variants specific for children. This could be due to different variants being associated with adiposity in childhood and adulthood, or due to stronger effects of some genetic variants on childhood adiposity than on adult adiposity [13, 14]. One example of such age dependency is that the association between genetic variants at the *FTO* locus and BMI varies by age [15, 16].

Maternal adiposity is a major risk predictor of childhood adiposity [17]. Genetic transmission from the mother to the child contributes largely to their phenotypic resemblance [1, 4, 18, 19]. However, specific maternal effects might also contribute, possibly via lifestyle factors, intrauterine growth environment, and shared environmental exposures during co-inhabitation [20–26]. These factors are inherently by themselves influenced by genetic and environmental factors, making it difficult to disentangle their individual contributions to childhood adiposity. While the evidence of specific maternal effects on childhood adiposity was ascertained in some studies [21, 27], other studies suggest little or no effect [28–30]. Intergenerational genetic studies using 83 adult BMI-associated SNPs found that maternal BMI-associated SNPs may contribute to the genetic link between prepregnancy BMI variation and offspring longitudinal weight [31] and that parent-of-origin effects may occur in a subset of these BMI predisposing SNPs [32]. Two intergenerational Mendelian randomization studies used adult BMI-associated genetic variants of the mother as instrumental variables to investigate if there is a causal effect of intrauterine exposure to greater maternal adiposity on offspring BMI [33, 34]. The results from both studies did not support a causal intrauterine effect of greater maternal BMI on childhood adiposity. Within the Danish National Birth Cohort (DNBC), we recently explored the effect of maternal BMI-associated genetic risk scores (GRS) on childhood overweight. The GRS was comprised of 941 genetic variants of adult BMI [12] splitted into maternal transmitted and non-transmitted genetic variants [35]. We found that the maternal transmitted GRS was associated with childhood overweight, but we did not find an association between the maternal non-transmitted GRS and child overweight [35], suggesting no specific influence of maternal adiposity as such.

We now performed an analysis in the same DNBC population of mother-child pairs but leveraged on child-specific GRSs in children, comprised of genetic variants identified in GWASs of children, as a more specific variable for childhood adiposity than the GRS for adult BMI used in the previous study. We utilized a weighted 26-SNP child BMI-GRS and a weighted 17-SNP child obesity-GRS. Our aim was to test whether the child GRSs for adiposity-related traits are associated with childhood adiposity independent of maternal BMI, and whether the associations are modified by maternal BMI.

## Methods and materials

### Study population

**Fig. 1:**
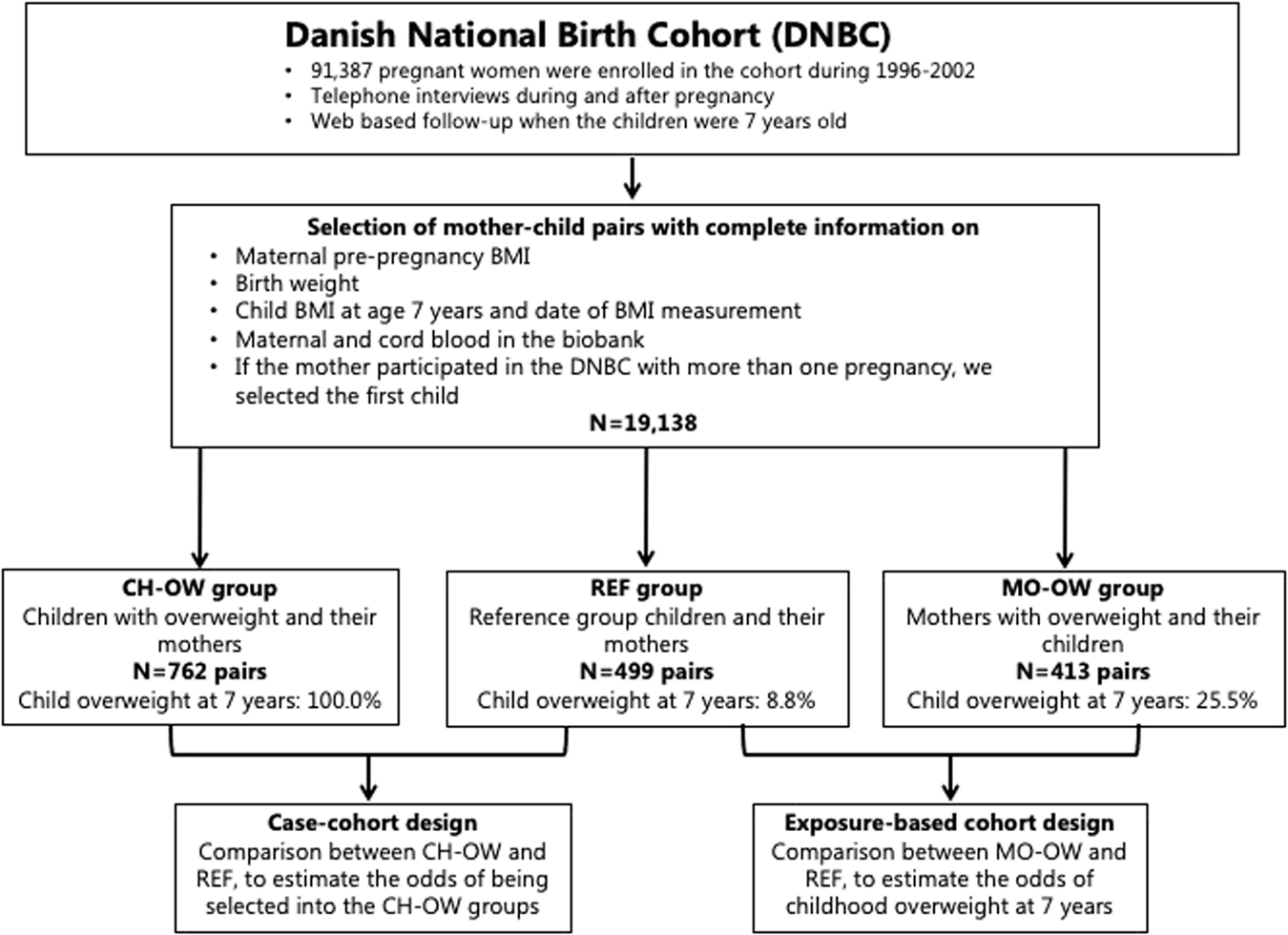
Participant flowchart and selection of children and mothers of the REF, CH-OW, and MO-OW groups. This selection provides the opportunity to perform a general survey of the study population (within REF analyses), and apply a case-cohort design (including the CH-OW and REF) and an exposure-based cohort design (including the MO-OW and REF) for childhood overweight.

The DNBC is a prospective birth cohort study, which enrolled 91,387 women across Denmark in 1996–2002 [36]. We have selected three sub-cohorts within the DNBC, as previously described and presented in Fig. 1 [35]. In brief, we selected three pairs of children and mothers with available BMI and genotype information for the present study: (1) randomly selected mothers and their children (REF, *N* = 499); (2) children with overweight at 7 years of age and their mothers (CH-OW, *N* = 762); (3) mothers with overweight and their children (MO-OW, *N* = 413). This selection provides the opportunity to perform a general survey of the study population (within the REF group), and apply a case-cohort design (including the CH-OW and REF groups) and an exposure-based cohort design (including the MO-OW and REF groups) for childhood overweight (Fig. 1). Each mother gave written informed consent at enrollment into the study. The genotyping of the mothers and children was approved by the Danish Ethical Committee (1-10-72-195-13 and 1-10-72-261-14). The study was conducted in accordance with the principles of the Declaration of Helsinki.

### Assessment of body mass index and definition of child overweight

BMI of children was based on weight and height information of the follow-up that was conducted when the children were around 7 years old. Sex- and age-specific SD scores for child BMI were derived. Childhood overweight at 7 years was defined based on the International Obesity Task Force (IOTF) [37]. BMI of the mothers was based on self-reported information on prepregnancy weight and height that was obtained during the telephone interview (conducted in Danish) in approximately gestational week 16 and converted to SD scores.

### Genotyping, SNP selection, and GRS construction

We previously described detailed information on genotyping procedures using Illumina platforms, calling of genotypes, and genotype quality control of the children, encompassing the exclusion of ethnic outliers, of the three groups (REF, CH-OW, and MO-OW) [35]. Genotype imputations were performed using the Haplotype Reference Consortium (release 1) at the server at the Sanger institute. The three groups were pooled together before imputation, with a total sample size of 1674. The imputation quality was considered high for all SNPs included in the current study (INFO > 0.77, Supplementary Tables 1–3). Four SNPs that were genome-wide significant in the childhood BMI or childhood obesity GWASs were missing in the imputation panel and excluded from our analysis without including proxy SNPs (rs184566112 from the child BMI GWAS; rs925494, rs2749808, and rs2540031 from the child obesity GWAS). The weighted child BMI-GRS (*N* = 26 independent SNPs) included 24 SNPs from the most recent EGG consortium GWAS meta-analysis of childhood BMI in 2020 [11], plus rs8092503 (near *RAB27B*) and rs3829849 (near *LMX1B*) that were identified in the 2016 EGG consortium meta-analysis of childhood BMI [9] (Supplementary Table 1). The weighted child obesity-GRS (*N* = 17 independent SNPs) included 16 SNPs from the most recent EGG consortium GWAS meta-analysis of childhood obesity [10], plus rs9568856 (near *OLFM4*) that was identified in the 2012 EGG consortium meta-analysis of childhood obesity [8] (Supplementary Table 2). We used the effect sizes (β) [9, 11] or odds ratios (OR) [8, 10] reported by the respective discovery GWAS studies to generate the weighted child BMI-GRS and child obesity-GRS. We also created an unweighted child adiposity-GRS by combining the number of adiposity-increasing alleles from overall 31 independent SNPs associated with childhood BMI and/or childhood obesity (Supplementary Table 3) to test whether this larger (combined) GRS may strengthen the results of the smaller child BMI-GRS and child obesity-GRS. For SNPs that are in LD with each other (*r*^2^ > 0.1), the ones derived from the largest sample sizes were included in the child adiposity-GRS. We standardized all GRSs into z-scores based on mean and SD of the respective GRS in the REF group.

### Statistical analyses

All analyses were conducted using R, version 3.6.3. We chose childhood overweight as outcome since the prevalence of childhood obesity at 7 years is so low in two of the three groups that the statistical power would be very limited and hence provide much more uncertain results than those based on childhood overweight (Supplementary Table 4). We used statistical models to confirm that maternal BMI and the child GRSs (child BMI-GRS and child obesity-GRS) were associated with child overweight, and to investigate if the child GRSs worked independent of maternal BMI. The latter was done, firstly, by adjusting the models for maternal BMI, and secondly, by interaction analyses testing whether the associations between child GRSs and child overweight status were modified by maternal BMI. The focus of this study was on the outcome child overweight, but we also applied within-group analyses of the REF group with continuous child BMI *z*-score as the outcome (linear regression). In the case-cohort analyses, we combined the CH-OW and REF groups to estimate the ORs for being selected into the CH-OW group (presented with 95% CI). In the exposure-based cohort design analysis, we combined the MO-OW and REF groups, to estimate the ORs for childhood overweight at 7 years as based on the IOTF criteria. In sensitivity analysis, we tested whether the larger (combined) child adiposity-GRS would strengthen the results of the smaller child BMI-GRS and child obesity-GRS. We did not adjust any of the models for age or sex of the child because, by construction, child BMI *z*-score is age- and sex-specific. An overview of the participant flowchart and the selection of children and mothers into the REF, CH-OW, and MO-OW are shown in Fig. 1.

## Results

### Phenotypic and GRS differences between the three sampling groups

As expected per sampling design, there was higher BMI and higher proportion of overweight in children of the CH-OW group, and among the mothers of the MO-OW and CH-OW groups, compared to the REF group (Supplementary Table 4).

The distribution of the child BMI/obesity/adiposity-increasing risk alleles, summarized in the three GRSs that were generated for the children are shown in Table 1. Children of the CH-OW and MO-OW groups carried a higher number of BMI-increasing, obesity-increasing, and adiposity-increasing risk alleles than the children of the REF group (Table 1). The correlations between the child GRSs and maternal BMI were low (*r* < 0.06) and nonsignificant in all three groups of mother-child dyads (Supplementary Table 5).

**Table 1:** Mean number of adiposity-increasing risk alleles of the three child GRSs (child BMI-GRS, child obesity-GRS, and child adiposity-GRS) within the three groups of children.

Child genetic risk scores (GRSs)	REF		CH-OW			MO-OW		
	*N*	Mean (SD)	*N*	Mean (SD)	P for difference between the CH-OW and the REF groups	*N*	Mean (SD)	*P* for difference between the MO-OW and the REF groups
Child BMI-GRS (*N* = 26 SNPs)								
Number of weighted BMI- increasing risk alleles	499	25.6 (2.5)	762	26.7 (2.6)	6.7E−15	413	26.2 (2.4)	1.7E−05
Child obesity-GRS (*N* = 17 SNPs)								
Number of weighted obesity-increasing risk alleles	499	15.1 (2.6)	762	16.0 (2.6)	1.4E−10	413	15.7 (2.5)	2.7E−04
Child adiposity-GRS (*N* = 31 SNPs)								
Number of unweighted adiposity-increasing risk alleles	499	30.7 (3.2)	762	32.0 (3.2)	2.2E−11	413	31.3 (3.1)	5.6E−03

The child BMI-GRS was comprised of 26 childhood BMI-associated variants, meaning that each child carried 52 alleles that were either BMI-increasing or decreasing. The child obesity-GRS was comprised of 17 childhood obesity-associated variants, meaning that each child carried 34 alleles that were either obesity risk-increasing or decreasing. The child adiposity-GRS was comprised of 31 adiposity-associated variants, meaning that each child carried 62 alleles that were either adiposity-increasing or decreasing. The GRSs are expressed as the number of BMI/obesity/adiposity-increasing alleles, respectively. We tested for differences in the GRSs between the groups of children in the CH-OW and MO-OW groups and the REF group using *t*-tests.

*CH-OW* group children with overweight and their mothers group, *GRS* genetic risk score*, MO-OW* group mothers with overweight and their children group, *REF group* reference group (randomly selected mothers and their children).

### Associations of child GRSs and maternal BMI with continuous child BMI *z*-score

In the REF group, maternal BMI and the child GRSs were approximately normally distributed and all positively associated with childhood BMI [maternal BMI: 0.28 SD (95% CI: 0.17; 0.39), child BMI-GRS: 0.17 SD (95% CI: 0.09; 0.26), child obesity-GRS: 0.22 SD (95% CI: 0.14; 0.30), Table 2]. Adjustment of the association between the child GRSs and child BMI for maternal BMI did not change the results materially (Table 2). There were no significant interactions between the GRSs and maternal BMI on child BMI (Table 2).

**Table 2:** Association results in the reference group (REF) on the outcome child BMI *z*-score as continuous trait (*N* = 495).

Determining variable	Child GRS			Maternal BMI			Child GRS × maternal BMI (interaction)		
	*β*	95% CI	*p*	*β*	95% CI	*p*	*β*	95% CI	*p*
Child BMI-GRS									
Unadjusted	0.17	(0.09,0.26)	4.4E−05	0.28	(0.17,0.39)	5.1E−07			
Adjusted	0.17	(0.09,0.25)	4.6E−05	0.28	(0.17,0.39)	5.2E−07	0.03	(0.00, 0.13)	0.59
Child obesity-GRS									
Unadjusted	0.22	(0.14,0.30)	1.2E−07	0.28	(0.17,0.39)	5.1E−07			
Adjusted	0.22	(0.14,0.30)	1.7E−07	0.27	(0.17,0.38)	7.1E−07	0.07	(0.01, 0.17)	0.24

Results from multiple linear regression analyses are given as estimated effects (β) in SD units (95% CI) of the various predictors on continuous child BMI *z*-score. We present unadjusted effects of the child GRSs and maternal BMI where child GRS and maternal BMI were used by themselves (one at a time), and adjusted effects where child GRS was adjusted for maternal BMI and maternal BMI was adjusted for child GRS from models without the interaction terms. Finally, the interaction effect is from the interaction model (child GRS × maternal BMI). The sample size was *N* = 495 for all reported analyses besides for the association with maternal BMI due to missing BMI information for one mother (*N* = 494).

*GRS* genetic risk score, *REF group,* reference group (randomly selected mothers and their children).

### Associations of child GRSs and maternal BMI with the odds of child overweight

#### Case-cohort design analysis of the CH-OW and REF groups

In the case-cohort design analysis, maternal BMI and the child GRSs were associated with increased odds for the children being in the CH-OW group [OR for maternal BMI: 2.01 (95% CI: 1.86; 2.17) OR for child BMI-GRS: 1.56 (95% CI: 1.47; 1.66), OR for child obesity-GRS 1.46 (95% CI: 1.37; 1.54) per SD unit, Table 3]. Adjustment of the association between the child GRSs and being in the CH-OW group for maternal BMI did not change the results materially (Table 3). There were no significant interactions between the GRSs and maternal BMI in these analyses (Table 3).

**Table 3:** Association results in the case-cohort design analysis on the outcome child overweight (*N* = 1261).

Determining variable	Child GRS			Maternal BMI			Child GRS × maternal BMI (interaction)		
	OR	95% CI	*p*	OR	95% CI	*p*	OR	95% CI	*p*
Child BMI-GRS									
Unadjusted	1.56	(1.47, 1.65)	7.2E−14	2.01	(1.86, 2.17)	4.2E−20			
Adjusted	1.55	(1.46, 1.65)	9.2E−13	2.00	(1.85, 2.16)	4.6E−19	0.97	(0.90, 1.04)	0.67
Child obesity-GRS									
Unadjusted	1.46	(1.37, 1.54)	3.5E−10	2.01	(1.86, 2.17)	4.2E−20			
Adjusted	1.43	(1.34, 1.52)	1.0E−08	1.98	(1.84, 2.14)	7.8E−19	0.95	(0.88, 1.03)	0.53

Results from logistic regression analyses are given as OR (95% CI) showing the effect of the various predictors on childhood overweight. In this case-cohort design-based analysis, we pooled the CH-OW and REF groups to estimate the odds of childhood overweight (odds for being selected into the CH-OW group). We present unadjusted effects of the child GRSs and maternal BMI where child GRS and maternal BMI were used by themselves (one at a time), and adjusted effects where child GRS was adjusted for maternal BMI and maternal BMI was adjusted for child GRS from models without the interaction terms. Finally, the interaction effect is from the interaction model (child GRS × maternal BMI). The sample size was *N* = 1261 for all reported analyses besides for the association with maternal BMI due to missing BMI information for one mother (*N* = 1260).

*CH-OW* group children with overweight and their mothers group, *GRS* genetic risk score, *OR* odds ratio, *REF* group reference group (randomly selected mothers and their children).

#### Exposure-based cohort design analysis of the MO-OW and REF groups

In the exposure-based cohort design analysis, we confirmed the overall pattern of the above estimated associations on the odds for childhood overweight. Maternal BMI and the child GRSs were associated with increased odds for childhood overweight [OR for maternal BMI: 1.46 (95% CI: 1.38; 1.54), OR for child BMI-GRS: 1.37 (95% CI: 1.25; 1.51), OR for child obesity-GRS 1.41 (95% CI: 1.28; 1.54) per SD unit, Table 4]. The odds for child overweight associated with the child GRSs decreased slightly by adjustment for maternal BMI as compared to the main effect of the association between the child GRSs and childhood overweight without adjusting for maternal effect (Table 4). There were also significant interactions between the child GRSs and maternal BMI on child overweight (*p* for interaction for child BMI-GRS × maternal BMI = 0.016, *p* for interaction for child obesity-GRS × maternal BMI = 0.005). The interaction effects were modest, suggesting 0.85–0.87-fold attenuation in the association between child GRSs or maternal BMI with ORs of overweight per SD unit increase in maternal BMI or child GRSs, respectively (Table 4).

**Table 4:** Association results from the exposure-based design-based analysis on the outcome child overweight (*N* = 912).

Determining variable	Child GRS			Maternal BMI			Child GRS × maternal BMI (interaction)		
	OR	95% CI	*p*	OR	95% CI	*p*	OR	95% CI	*p*
Child BMI-GRS									
Unadjusted	1.37	(1.25, 1.50)	8.0E−04	1.46	(1.38, 1.54)	2.0E−12			
Adjusted	1.28	(1.16, 1.41)	1.1E−02	1.44	(1.36, 1.52)	1.7E−11	0.87	(0.82, 0.92)	0.016
Child obesity-GRS									
Unadjusted	1.41	(1.28, 1.54)	2.1E−04	1.46	(1.38, 1.54)	2.0E−12			
Adjusted	1.32	(1.20, 1.46)	3.4E−03	1.44	(1.36, 1.52)	1.9E−11	0.85	(0.80, 0.90)	0.005

Results from logistic regression analyses are given as OR (95% CI) showing the effect of the various predictors on childhood overweight. In this exposure-based analysis, we pooled the MO-OW and REF groups to estimate the odds of childhood overweight (child with overweight at 7 years). We present unadjusted effects of the child GRSs and maternal BMI where child GRS and maternal BMI were used by themselves (one at a time), and adjusted effects where child GRS was adjusted for maternal BMI and maternal BMI was adjusted for child GRS from models without the interaction terms. Finally, the interaction effect is from the interaction model (child GRS × maternal BMI). To make the estimated effects for the various interaction variable levels well-defined one needs the corresponding estimates for the single variable-effects (often called the main effects, i.e., now the respective effect given that the complementary interaction variable equals zero). In the models including the interaction terms, the OR (95% CI) for child GRSs (given maternal BMI being zero) were for child BMI-GRS: 1.74 (1.48, 2.04), child obesity-GRS: 1.88 (1.60, 2.21); and the OR (95% CI) for maternal BMI (given child GRSs being zero) were child BMI-GRS: 1.51 (1.42, 1.60), child obesity-GRS: 1.52 (1.44, 1.62). Sample size was *N* = 912 for all reported analyses besides for the models including maternal BMI due to missing BMI information for one mother (*N* = 911).

*GRS* genetic risk score, *MO-OW group* mothers with overweight and their children group, *OR* odds ratio, *REF group* reference group (randomly selected mothers and their children).

#### Sensitivity analysis applying a combined unweighted child adiposity-GRS

When applying the larger combined unweighted child adiposity-GRS, we confirmed the overall pattern of the estimated associations between child GRSs and continuous child BMI *z*-score/odds of child overweight. However, the effect sizes seen for the child adiposity-GRS were generally slightly weaker (Supplementary Table 6).

## Discussion

The present study confirmed that both maternal BMI and child-specific GRSs for adiposity-related traits contribute to childhood adiposity. We explored whether maternal phenotypic BMI influences the association between child GRSs and childhood adiposity by adjusting the association between child GRSs and childhood adiposity for maternal BMI as a covariate, and by testing for child GRS × maternal BMI interaction. The results highlight that child GRSs associate with childhood adiposity independent of maternal BMI, which appears not to modify the effect of child GRSs for adiposity-related traits on childhood adiposity within the central range of maternal BMI. However, we found in children of mothers with overweight that the effect of child GRSs or maternal BMI on childhood overweight was less strong the higher the maternal BMI or child GRSs, respectively.

Limitations of this study include using self-reported information of maternal and child weight and height, reported by the mothers, which may introduce recall bias especially in the phenotypic extremes [38]. Information based on reporting by others is generally less accurate and less reliable [39], and therefore we cannot exclude that there may be greater random and possibly systematic error in the reporting of child weight and height by their mother. However, weight and height data in the 7-year follow-up telephone interview were validated against measured height and weight from school health records in a sub-sample of the DNBC, and no trend towards increasing difference in weight or height with increasing averages of weight or height between the reported and objectively measured values were found, suggesting there was no systematic error [40]. We constructed the child GRSs assuming additive genetic effects for all included GWAS-identified common genetic variants, but cannot preclude possible nonadditive effects. The weighted child obesity-GRS included SNPs that were selected from a trans-ancestral GWAS meta-analysis of childhood obesity [10]. However, all selected SNPs were associated with childhood obesity in the European population in the study by Bradfield et al. [10]. In this study, we only focused on maternal phenotypic BMI effects on childhood adiposity, independent from the genetic transmission, but without addressing possible paternal effects. In addition to assuming that maternal BMI may be more important than paternal BMI in the present context, we were also concerned about the possibly poorer quality of maternally reported paternal height and weight at child age 18 months [35].

A major strength of the present study is the application of the child GRSs based on common genetic variants identified in large GWAS analysis that were specifically conducted in children, showing strong associations with child BMI and overweight in our study. We also defined a child adiposity-GRS by combining genetic variants identified for child BMI and child obesity to test whether this larger GRS would have the ability to strengthen the results of the smaller child BMI-GRS and child obesity-GRS. The overall weaker effect sizes seen for the child adiposity-GRS are likely due to the fact that, in contrast to both the child BMI-GRS and the child obesity-GRS, this GRS was not weighted by the effect sizes (*β* or OR, respectively) of the included genetic variants as reported by the respective discovery GWAS, which was not immediately possible when combining them. Another strength of our study is the sampling design based on mother-child pairs with extreme BMI conditions within the large DNBC: mothers with overweight and their children, children with overweight and their mothers and a reference group of randomly selected mothers and their children. The statistical power of the applied case-cohort and exposure-based cohort design is demonstrated by the precision of the estimates. Furthermore, chosing BMI and overweight at around age 7 years as outcome avoids challenges implicit in the childhood pattern of growth in BMI, which does not follow a linear trajectory from birth until adolescence. Thus, childhood BMI is characterized by a rapid increase from birth to around 9 months reaching adiposity peak, a decline from 9 months to 5 or 6 years of age, followed by a steady increase until adolescence during a phase called the “adiposity rebound”, and finally decelerated growth towards adulthood [41]. By using data of children at around 7 years of age and their age- and sex-specific BMI *z*-scores in our study, we avoided the adiposity decline period before adiposity rebound as well as the specific pubertal effects on adiposity, where there are considerable differences in the growth curves between boys and girls.

We found an attenuation of the effect of the child GRSs on childhood adiposity with increasing maternal BMI in mothers with overweight, confirming our previous observation [35]. The interaction term for the product of child GRSs and maternal BMI indicates an attenuation of the effect of maternal BMI on childhood adiposity with increasing child GRSs. This finding is not supportive of the developmental overnutrition hypothesis, and thereby consistent with the findings of two intergenerational Mendelian randomization studies that did not find evidence to support a strong causal intrauterine effect of greater maternal BMI resulting in greater offspring adiposity [33, 34]. One possible explanation of our finding could be that excessive maternal BMI is an indicator of stronger, shared though unspecified, environmental influences that may dilute the genetic effects on childhood BMI and overweight (reviewed in [42]). We observed these child GRS × maternal BMI interactions only in the exposure-based cohort, likely due to the broader range of maternal BMI ranging from normal BMI (reference group) to high BMI (group of mothers with overweight). We suggest this means that various genetic and/or environmental factors may contribute to the overweight status of the selected mothers and the risk of overweight of their children that were not probed by the child GRSs for adiposity-related traits. While we assumed a linear effect of both maternal BMI and the GRSs on childhood overweight in the analyses, we cannot preclude nonlinear effects. Furthermore, there may be both negative and positive confounding of both the general influences of maternal BMI as well as of the observed attenuation of the child GRS at high maternal BMI, which may be addressed in future studies.

Recently, a large GRS for childhood adiposity, including 295 genetic variants, was generated in UK Biobank, in which adult participants provided questionnaire-derived data about their relative body size at 10 years of age [43]. Such questionnaire-based, retrospective assessment of childhood body size is prone to recall and misclassification bias, but the GRS based on these 295 SNPs for childhood adiposity was validated using measured BMI data in 12-year-old adolescents and adults from the Norwegian HUNT study [44]. Notably, both the UK Biobank and the HUNT study found a considerable overlap between genetic variants associated with BMI in childhood and adulthood, but also considerable differences [43, 44]. The predictive performance of the 295-SNP child GRS was better in adolescents and early adulthood, and the predicitive performance of the 557-SNP adult GRS was better in adulthood [44]. This supports our use of the child GRSs as more specific variables for childhood adiposity estimated at 7 years despite the overlap with genetic variants derived from the GWAS of adults. Although the 295-SNP child GRS for child adiposity includes a larger number of variants and may thus explain a somewhat larger variance of childhood BMI and overweight than the GRSs applied in our study, we do not expect to find a different effect of maternal BMI in terms of influencing or modifying the effect of a larger childhood GRS. Nevertheless, replicating our findings with the use of large sample-based polygenic risk scores—together with a better understanding of the causality of the individual genetic loci that the SNPs are indicators of, and the possible interaction with them—should ideally be done in future projects.

The strength of the association of child GRSs with adiposity-related traits is too weak to be useful for clinical risk prediction of child overweight. Moreover, applying GRS approaches to overweight risk assessment offers little in the absence of more detailed information, both about the specific effects of the predisposing SNPs, whether or not captured by the GRS, and about the lifestyle and environment of the individual mother-child dyads [45]. However, the child GRSs constitute a useful tool that informs etiological research of childhood overweight.

In conclusion, we confirm that child GRSs for adiposity-related traits and maternal BMI are both associated with childhood adiposity. We did not find evidence that maternal BMI within the normal range influences or modifies the effect of child GRSs for adiposity-related traits on childhood adiposity, suggesting that GRSs for childhood adiposity operate independently of the BMI of their mothers. However, in children of mothers with overweight the effect of child GRSs on childhood overweight was attenuated.

## Supplementary information

Supplemental Appendix

## Data Availability

Relevant data for the present study are within the article and its Supporting Information files. R code is available from the authors upon reasonable request. Data are available from the DNBC and can be requested through the steering committee of the study who can be contacted at dnbc-research@ssi.dk. More information regarding access to data can be found on the DNBC website https://www.dnbc.dk/access-to-dnbc-data.
