# Supplementary material for: Do genetic risk scores for childhood adiposity operate independent of BMI of their mothers?
Source: Int J Obes (Lond). 2021 May 28;45(9):2006–15. doi: 10.1038/s41366-021-00869-4 (PMC8380541; doi:10.1038/s41366-021-00869-4)
Supplement: Supplementary file 1 — Supplemental Appendix [file 41366_2021_869_MOESM1_ESM.docx]

**Supplemental Appendix**

**Supplemental Table 1. Information of the 26 SNPs included in the weighted child BMI-GRS.**

| SNP | Chr | Position | Locus | EA | OA | EAF | Beta | GWAS^*^ | INFO^#^ |
| --- | --- | --- | --- | --- | --- | --- | --- | --- | --- |
| rs61765651 | 1 | 72754314 | NEGR1 | C | T | 0.83 | 0.047 | V(2020) | 0.98 |
| rs12042908 | 1 | 74997762 | FPGT-TNNI3K | A | G | 0.46 | 0.064 | V(2020) | 0.88 |
| rs144376234 | 1 | 110114504 | GNAI3 | T | C | 0.04 | 0.111 | V(2020) | 0.81 |
| rs543874 | 1 | 177889480 | SEC16B | G | A | 0.19 | 0.075 | V(2020) | 0.95 |
| rs1094647 | 1 | 205655378 | SLC45A3 | G | A | 0.55 | 0.038 | V(2020) | 0.97 |
| rs62107261 | 2 | 422144 | FAM150B | T | C | 0.95 | 0.121 | V(2020) | 0.83 |
| rs939584 | 2 | 621558 | TMEM18 | T | C | 0.83 | 0.092 | V(2020) | 0.99 |
| rs11676272 | 2 | 25141538 | ADCY3 | G | A | 0.46 | 0.071 | V(2020) | 0.94 |
| rs114670539 | 2 | 207064335 | GPR1 | T | C | 0.05 | 0.088 | V(2020) | 0.89 |
| rs12641981 | 4 | 45179883 | GNPDA2 | T | C | 0.44 | 0.045 | V(2020) | 1.00 |
| rs13107325 | 4 | 103188709 | SLC39A8 | T | C | 0.07 | 0.082 | V(2020) | 0.80 |
| rs7719067 | 5 | 153538241 | GALNT10 | A | G | 0.43 | 0.036 | V(2020) | 0.99 |
| rs3130622 | 6 | 31592524 | PRRC2A | C | G | 0.18 | 0.049 | V(2020) | 1.00 |
| rs2076308 | 6 | 50791640 | TFAP2B | C | G | 0.19 | 0.058 | V(2020) | 0.98 |
| rs62500888 | 8 | 28061823 | ELP3 | A | G | 0.57 | 0.037 | V(2020) | 0.93 |
| rs3829849 | 9 | 129390800 | LMX1B | T | C | 0.36 | 0.041 | F(2016) | 0.78 |
| rs56133711 | 11 | 27723334 | BDNF | A | G | 0.24 | 0.056 | V(2020) | 1.00 |
| rs11030391 | 11 | 28644626 | METTL15 | A | G | 0.63 | 0.036 | V(2020) | 0.97 |
| rs11215427 | 11 | 115093438 | CADM1 | G | C | 0.74 | 0.039 | V(2020) | 0.97 |
| rs7138803 | 12 | 50247468 | BCDIN3D | A | G | 0.37 | 0.072 | V(2020) | 0.84 |
| rs4477562 | 13 | 54104968 | LINC00558 | T | C | 0.13 | 0.065 | V(2020) | 0.93 |
| rs114285994 | 16 | 19935763 | GPRC5B | G | A | 0.87 | 0.063 | V(2020) | 0.95 |
| rs17817449 | 16 | 53813367 | FTO | G | T | 0.40 | 0.069 | V(2020) | 0.99 |
| rs8092503 | 18 | 52479487 | RAB27B | G | A | 0.27 | 0.045 | F(2016) | 0.94 |
| rs184566112 | 18 | 55943926 | NEDD4L | A | T | 0.84 | 0.057 | V(2020) | excluded |
| rs571312 | 18 | 57839769 | MC4R | A | C | 0.23 | 0.052 | V(2020) | 1.00 |
| rs76227980 | 18 | 58036384 | MC4R | C | T | 0.98 | 0.140 | V(2020) | 0.80 |

^*^Overall, 24 SNPs were identified in the discovery GWAS by Vogelezang et al. in 2020, while 2 SNPs were identified by Felix et al. in 2016. Estimated effects (β:s) for the calculation of the weighted child BMI-GRS were taken from the indicated discovery GWAS.

^#^ INFO refers to the imputation quality of the SNPs of the children of the Danish National Birth Cohort; or indicates whether the SNP was excluded.

*Abbreviations. Chr: Chromosome; EA: Effect allele; EAF: Effect allele frequency; F(2016): Felix et al. in 2016; OA: other allele; SNP: Single nucleotide polymorphism; V(2020): Vogelezang et al. in 2020*

**Supplemental Table 2. Information of 17 SNPs included in the weighted child obesity-GRS.**

| SNP | Chr | Position | Locus | EA | OA | EAF | Beta | GWAS^*^ | INFO^#^ |
| --- | --- | --- | --- | --- | --- | --- | --- | --- | --- |
| rs10493544 | 1 | 74983835 | TNNI3K | T | C | 0.46 | 0.140 | B(2019) | 0.88 |
| rs539515 | 1 | 177889025 | SEC16B | A | C | 0.79 | -0.180 | B(2019) | 0.95 |
| rs62104180 | 2 | 466003 | TMEM18 | A | G | 0.03 | -0.320 | B(2019) | 0.82 |
| rs7579427 | 2 | 631183 | TMEM18 | A | C | 0.85 | 0.210 | B(2019) | 0.99 |
| rs4077678 | 2 | 25122840 | ADCY3 | C | G | 0.44 | -0.140 | B(2019) | 0.94 |
| rs114670539 | 2 | 207064335 | GPR1 | T | C | 0.04 | 0.260 | B(2019) | 0.89 |
| rs925494 | 4 | 45187622 | GNPDA2 | T | C | 0.35 | 0.100 | B(2019) | excluded |
| rs2053682 | 5 | 170599327 | RANBP17 | A | C | 0.68 | 0.090 | B(2019) | 0.96 |
| rs2206277 | 6 | 50798526 | TFAP2B | T | C | 0.19 | 0.140 | B(2019) | 0.98 |
| rs10224397 | 7 | 93269367 | CALCR | A | G | 0.54 | 0.090 | B(2019) | 0.94 |
| rs17309874 | 11 | 27667236 | BDNF | A | G | 0.18 | 0.120 | B(2019) | 0.98 |
| rs10835310 | 11 | 28355657 | METTL15 | T | C | 0.45 | 0.100 | B(2019) | 0.95 |
| rs7132908 | 12 | 50263148 | FAIM2 | A | G | 0.30 | 0.150 | B(2019) | 0.86 |
| rs9568856 | 13 | 54064981 | OLFM4 | A | G | 0.23 | 0.093 | B(2012) | 0.91 |
| rs2540031 | 16 | 4017567 | ADCY9 | A | T | 0.47 | 0.080 | B(2012) | excluded |
| rs56094641 | 16 | 53806453 | FTO | A | G | 0.71 | -0.210 | B(2019) | 0.99 |
| rs2740752 | 17 | 46664608 | HOXB5 | T | C | 0.80 | 0.110 | B(2019) | 0.92 |
| rs6567160 | 18 | 57829135 | MC4R | T | C | 0.79 | -0.150 | B(2019) | 0.98 |
| rs2749808 | 20 | 54149014 | CBLN4 | T | C | 0.64 | -0.100 | B(2019) | excluded |
| rs1437206 | 20 | 54482276 | CBLN4 | T | C | 0.32 | -0.100 | B(2019) | 1.00 |

^*^Overall, 16 SNPs were identified in the discovery GWAS by Bradfield et al. in 2019, while 1 SNP was identified by Bradfield et al. in 2012. Estimated effects (β:s) for the calculation of the weighted obesity BMI-GRS were taken from the indicated discovery GWAS.

^#^ INFO refers to the imputation quality of the SNPs of the children of the Danish National Birth Cohort; or indicates whether the SNP was excluded.

*Abbreviations. B(2019): Bradfield et al. in 2019; B(2012): Bradfield at al. in 2012; Chr: Chromosome; EA: Effect allele; EAF: Effect allele frequency; OA: other allele; SNP: Single nucleotide polymorphism*

**Supplemental Table 3. Information of the 31 SNPs included in the unweighted child adiposity-GRS.**

| SNP | Chr | Position | Locus | EA | OA | EAF | GWAS^*^ | INFO^#^ |
| --- | --- | --- | --- | --- | --- | --- | --- | --- |
| rs61765651 | 1 | 72754314 | NEGR1 | C | T | 0.83 | V(2020) | 0.98 |
| rs12042908 | 1 | 74997762 | FPGT-TNNI3K | A | G | 0.46 | V(2020) | 0.88 |
| rs144376234 | 1 | 110114504 | GNAI3 | T | C | 0.04 | V(2020) | 0.81 |
| rs543874 | 1 | 177889480 | SEC16B | G | A | 0.19 | V(2020) | 0.95 |
| rs1094647 | 1 | 205655378 | SLC45A3 | G | A | 0.55 | V(2020) | 0.97 |
| rs62107261 | 2 | 422144 | FAM150B | T | C | 0.95 | V(2020) | 0.83 |
| rs939584 | 2 | 621558 | TMEM18 | T | C | 0.83 | V(2020) | 0.99 |
| rs11676272 | 2 | 25141538 | ADCY3 | G | A | 0.46 | V(2020) | 0.94 |
| rs114670539 | 2 | 207064335 | GPR1 | T | C | 0.05 | V(2020) | 0.89 |
| rs12641981 | 4 | 45179883 | GNPDA2 | T | C | 0.44 | V(2020) | 1.00 |
| rs13107325 | 4 | 103188709 | SLC39A8 | T | C | 0.07 | V(2020) | 0.80 |
| rs7719067 | 5 | 153538241 | GALNT10 | A | G | 0.43 | V(2020) | 0.99 |
| rs2053682 | 5 | 170599327 | RANBP17 | A | C | 0.68 | B(2019) | 0.96 |
| rs3130622 | 6 | 31592524 | PRRC2A | C | G | 0.18 | V(2020) | 1.00 |
| rs2076308 | 6 | 50791640 | TFAP2B | C | G | 0.19 | V(2020) | 0.98 |
| rs10224397 | 7 | 93269367 | CALCR | A | G | 0.54 | B(2019) | 0.94 |
| rs62500888 | 8 | 28061823 | ELP3 | A | G | 0.57 | V(2020) | 0.93 |
| rs3829849 | 9 | 129390800 | LMX1B | T | C | 0.36 | F(2016) | 0.78 |
| rs56133711 | 11 | 27723334 | BDNF | A | G | 0.24 | V(2020) | 1.00 |
| rs11030391 | 11 | 28644626 | METTL15 | A | G | 0.63 | V(2020) | 0.97 |
| rs11215427 | 11 | 115093438 | CADM1 | G | C | 0.74 | V(2020) | 0.97 |
| rs7138803 | 12 | 50247468 | BCDIN3D | A | G | 0.37 | V(2020) | 0.84 |
| rs9568856 | 13 | 54064981 | OLFM4 | A | G | 0.16 | B(2012) | 0.91 |
| rs4477562 | 13 | 54104968 | LINC00558 | T | C | 0.13 | V(2020) | 0.93 |
| rs114285994 | 16 | 19935763 | GPRC5B | G | A | 0.87 | V(2020) | 0.95 |
| rs17817449 | 16 | 53813367 | FTO | G | T | 0.40 | V(2020) | 0.99 |
| rs2740752 | 17 | 46664608 | HOXB5 | T | C | 0.80 | B(2019) | 0.92 |
| rs8092503 | 18 | 52479487 | RAB27B | G | A | 0.27 | F(2016) | 0.94 |
| rs571312 | 18 | 57839769 | MC4R | A | C | 0.23 | V(2020) | 1.00 |
| rs76227980 | 18 | 58036384 | MC4R | C | T | 0.98 | V(2020) | 0.80 |
| rs1437206 | 20 | 54482276 | CBLN4 | T | C | 0.32 | B(2019) | 1.00 |

^*^Discovery GWAS indicates from which GWAS the SNP was taken to base the adiposity-GRS on.

^#^ INFO refers to the imputation quality of the SNPs of the children of the Danish National Birth Cohort.

*Abbreviations. B(2019): Bradfield et al. in 2019; B(2012): Bradfield at al. in 2012; Chr: Chromosome; EA: Effect allele; EAF: Effect allele frequency; F(2016): Felix et al. in 2016; OA: other allele; SNP: Single nucleotide polymorphism; V(2020): Vogelezang et al. in 2020*

**Supplemental Table 4. Study characteristics of the REF, CH-OW and MO-OW groups selected within the Danish National Birth Cohort**

|  | REF | | CH-OW | | p for difference between the CH-OW and REF groups | MO-OW | | p for difference between the MO-OW and REF groups |
| --- | --- | --- | --- | --- | --- | --- | --- | --- |
|  | N | Mean (SD) or % | N | Mean (SD) or % |  | N | Mean (SD) or % |  |
| **Child BMI characteristics** |  |  |  |  |  |  |  |  |
| BMI (kg/m^2^) | 499 | 15.6 (1.6) | 762 | 20.1 (2.0) | <0.001 | 413 | 16.8 (2.3) | <0.001 |
| BMI (SD-units) |  | 0.0 (1.00) |  | 2.1 (0.5) |  |  | 0.6 (1.1) |  |
| Overweight (%) |  |  |  |  |  |  |  |  |
| *No* |  | 91.2 |  | 0.0 | <0.001 |  | 74.8 | <0.001 |
| *Yes* |  | 8.8 |  | 100.0 |  |  | 25.2 |  |
| Obesity (%) |  |  |  |  |  |  |  |  |
| *No* |  | 99.2 |  | 93.0 | 0.07 |  | 71.6 | <0.001 |
| *Yes* |  | 0.8 |  | 7.0 |  |  | 28.4 |  |
| **Maternal BMI characteristics** |  |  |  |  |  |  |  |  |
| BMI (kg/m^2^) | 498 | 23.1 (3.2) | 762 | 25.1 (3.7) | <0.001 | 413 | 37.1 (3.3) | <0.001 |
| BMI (SD-units) |  | -0.1 (0.8) |  | 0.4 (0.9) | <0.001 |  | 3.2 (0.8) | <0.001 |
| Overweight (%) |  |  |  |  |  |  |  |  |
| *No* |  | 76.1 |  | 46.6 | <0.001 |  | 0.0 | <0.001 |
| *Yes* |  | 23.9 |  | 53.4 |  |  | 100.0 |  |
| **Other characteristics** |  |  |  |  |  |  |  |  |
| Child gender (%) | 499 |  | 762 |  |  | 413 |  |  |
| *Female* |  | 48.9 |  | 49.6 | 0.85 |  | 48.9 | 0.99 |
| *Male* |  | 51.1 |  | 50.4 |  |  | 51.1 |  |
| Child age (years) | 499 | 7.1 (0.3) | 762 | 7.1 (0.3) | 0.50 | 413 | 7.1 (0.2) | 0.92 |
| Maternal age (years) | 499 | 30.5 (4.1) | 762 | 30.6 (4.2) | 0.81 | 413 | 30.1 (3.9) | 0.10 |

Child BMI characteristics were from 7 years of age, and maternal BMI characteristics were from pre-pregnancy. We tested for differences in continuous characteristics between the groups of children and mothers at the extremities of the BMI distribution (CH-OW and MO-OW groups) and the REF group using t-tests and for differences in binary characteristics using two-proportions z-test integrated into the “prop.test” function in R software.

*Abbreviations. CH-OW group: Children with overweight and their mothers group; MO-OW group: Mothers with overweight and their children group; REF group: Reference group (randomly selected mothers and their children)*

**Supplemental Table 5. Pearson correlations between the three child GRSs and maternal BMI in the three groups of mother-child dyads**

| Group | Child GRS | Correlation with maternal BMI (r) | p |
| --- | --- | --- | --- |
| REF | Child BMI-GRS | 0.019 | 0.67 |
| REF | Child obesity-GRS | 0.039 | 0.39 |
| REF | Child adiposity-GRS | 0.053 | 0.24 |
| CH-OW | Child BMI-GRS | 0.014 | 0.69 |
| CH-OW | Child obesity-GRS | 0.048 | 0.19 |
| CH-OW | Child adiposity-GRS | 0.004 | 0.92 |
| MO-OW | Child BMI-GRS | 0.005 | 0.92 |
| MO-OW | Child obesity-GRS | 0.036 | 0.46 |
| MO-OW | Child adiposity-GRS | -0.031 | 0.53 |

*Abbreviations. CH-OW group: Children with overweight and their mothers group groups; GRS: Genetic risk score; MO-OW group: Mothers with overweight and their children; REF group: Reference group (randomly selected mothers and their children).*

**Supplemental Table 6. Sensitivity analysis of the association results by applying the unweighted child-adiposity GRS.**

| Statistical model | Child adiposity-GRS | | | Maternal BMI | | | Child adiposity-GRS×maternal BMI (interaction) | | |
| --- | --- | --- | --- | --- | --- | --- | --- | --- | --- |
|  | β/OR | 95% CI | p | β/OR | 95% CI | p | β/OR | 95% CI | p |
| **Association results in the reference group on the outcome child BMI z-score as continuous trait (in β**) | | | | | | | | | |
| Unadjusted | 0.15 | (0.07,0.24) | 3.1E-04 | 0.28 | (0.17,0.39) | 5.1E-07 |  |  |  |
| Adjusted | 0.14 | (0.06,0.22) | 6.1E-04 | 0.27 | (0.16,0.38) | 9.9E-07 | 0.01 | (0.00,0.11) | 0.92 |
| **Association results in the case-cohort design analysis on the outcome child overweight (in OR)** | | | | | | | | | |
| Unadjusted | 1.49 | (1.40,1.58) | 5.3E-11 | 2.01 | (1.86,2.17) | 4.2E-20 |  |  |  |
| Adjusted | 1.48 | (1.39,1.57) | 7.5E-10 | 2.00 | (1.85,2.16) | 4.0E-19 | 0.91 | (0.84,0.99) | 0.24 |
| **Association results in the exposure-based cohort design analysis on the outcome child overweight (in OR)** | | | | | | | | | |
| Unadjusted | 1.38 | (1.26,1.51) | 6.0E-04 | 1.46 | (1.38,1.54) | 2.0E-12 |  |  |  |
| Adjusted | 1.34 | (1.21,1.47) | 2.9E-03 | 1.45 | (1.37,1.53) | 7.0E-12 | 0.88 | (0.83,0.93) | 0.028 |

Results from multiple linear regression analyses are given as estimated effects (β) in SD-units (95% CI) of the unweighted child adiposity-GRS predictor on continuous child BMI z-score in the reference group (N=495). Results from logistic regression analyses are given as OR (95% CI) showing the effect of the unweighted child adiposity-GRS predictor on childhood overweight by applying a case-cohort design analysis (N=1,261) and a exposure-based cohort design analysis (N=912). We present unadjusted effects of the unweighted child adiposity-GRS and maternal BMI where unweighted child adiposity-GRS and maternal BMI were used by themselves (one at a time), and adjusted effects where unweighted child adiposity-GRS was adjusted for maternal BMI and maternal BMI was adjusted for unweighted child adiposity-GRS from models without the interaction terms. Finally, the interaction effect is from the interaction model (unweighted child adiposity-GRS×maternal BMI). To make the estimated effects for the interaction variable levels well-defined one needs the corresponding estimates for the single variable-effects (often called the main effects, i.e. now the respective effect given that the complementary interaction variable equals zero). In the model of the exposure-based cohort design analysis that includes the interaction term, the OR (95% CI) for unweighted child adiposity-GRS (given maternal BMI being zero) was 1.75 (1.49,2.05); and the OR (95% CI) for maternal BMI (given unweighted child adiposity-GRS being zero) was 1.50 (1.42,1.59).

*Abbreviations. GRS: Genetic risk score; OR: odds ratio*
